# Transperitoneal Laparoscopic Pyelopyelostomy for Retrocaval Ureter without Excision of the Retrocaval Segment: Experience on Three Cases

**DOI:** 10.1155/2016/5709134

**Published:** 2016-06-14

**Authors:** Y. El Harrech, O. Ghoundale, E. H. Kasmaoui, D. Touiti

**Affiliations:** ^1^Department of Urology, Military Hospital Avicenne, 40000 Marrakech, Morocco; ^2^Kasmaoui Medical Office, 90000 Tangier, Morocco

## Abstract

*Introduction*. Retrocaval ureter is a rare congenital anomaly. Open surgery was the classic treatment for this condition. Laparoscopy is currently an admitted procedure to treat many urological diseases. The objective of our study is to present our experience and discuss the safety and the feasibility of transperitoneal laparoscopic pyelopyelostomy for treatment of retrocaval ureter (RCU).* Materials and Methods*. Three symptomatic patients underwent laparoscopic repair for RCU in our department. The diagnosis was suspected on the computed tomography scan (CT) and confirmed on ascending pyelography. After placement of a JJ stent, and, using the transperitoneal approach, the retro peritoneum was exposed; the ureter was identified in both sides of the vena cava. The retrocaval segment was entirely mobilized and pulled from behind of the vena cava after section of renal pelvis. A pyelopyelostomy was done in a normal anatomic position.* Results*. All operations were achieved laparoscopically without conversion to open surgery. The mean operative time was 140 minutes (110–190). No intraoperative complication occurred. Blood loss was less than 50 mL in all patients. The mean hospital stay was 5 days (4–6 days). All patients were symptom-free after surgery and had reduction of hydronephrosis in control imagery.* Conclusion*. Laparoscopy seems safe, feasible, and reproducible in managing retrocaval ureter.

## 1. Introduction

Retrocaval ureter is a rare congenital anomaly reported the first time by Hochstetter in 1893 [1]. Open surgery was the classic treatment of this pathology with excision of the retrocaval segment, anteposition, and ureteroureteral or ureteropelvic reanastomosis [2, 3].

The development of laparoscopy and performing skills for urologists have permitted enlargement of indications for this procedure in urology field. Laparoscopic dismembered pyeloplasty became the treatment of choice of ureteropelvic junction obstruction (UPJO) with equivalent results compared to open surgery, with less postoperative pain and shorter convalescence [4, 5].

Because of the rarity of their congenital disease, few cases of laparoscopic repair are reported. We present three cases of retrocaval ureter corrected by transperitoneal laparoscopy.

## 2. Materials and Methods

Between February 2012 and June 2015, three patients (2 females and one male) underwent laparoscopic repair for RCU in our department. Mean age was 36 years (range 18–48). All patients were symptomatic with in all cases a history of intermittent moderate right-side flank pain. One female patient had repeated episodes of acute pyelonephritis on the same side.

Abdominal ultrasound demonstrated right renal hydronephrosis in all patients. Urography showed medial displacement of the right JJ catheter. A computed tomography scan (CT) suspected the presence of a retrocaval ureter. Diuretic renography with ^99m^Tc-DTPA showed significant obstruction in one patient.

After informed consent, the patients had laparoscopic pyelopyelostomy as described below.

**Figure 1:**
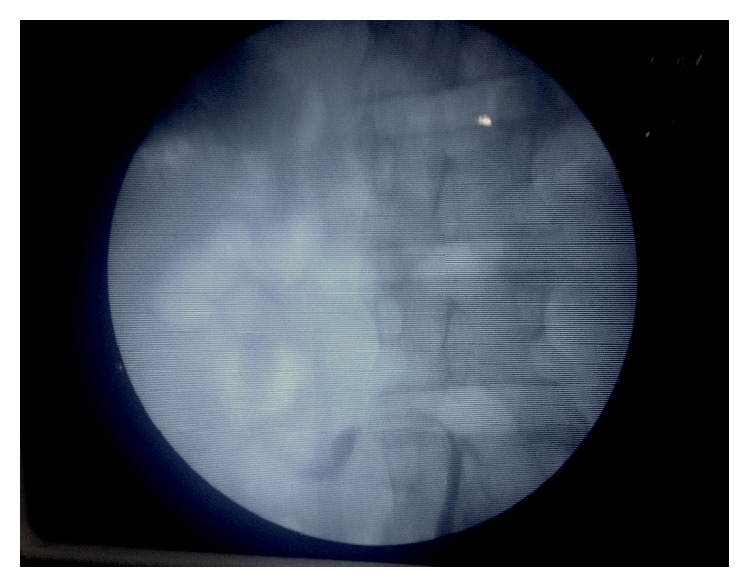
Typical image in the form of a hook or S-shaped on ascending pyelography.

*Surgical Technique.* Operation was performed under general anaesthesia. The patient was first placed in the lithotomy position and underwent right ascending pyelography to confirm the diagnosis (typical image in the form of a hook or S-shaped) (Figure 1). A right JJ catheter was then placed. After that, the patient was placed in right-side-up flank position and a transperitoneal approach was used.

**Figure 2:**
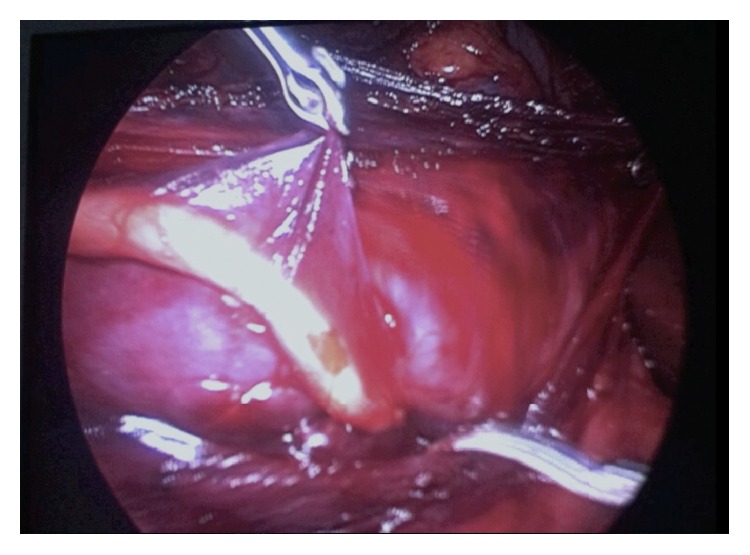
The ureter crossing left side of vena cava and disappearing behind it.

**Figure 3:**
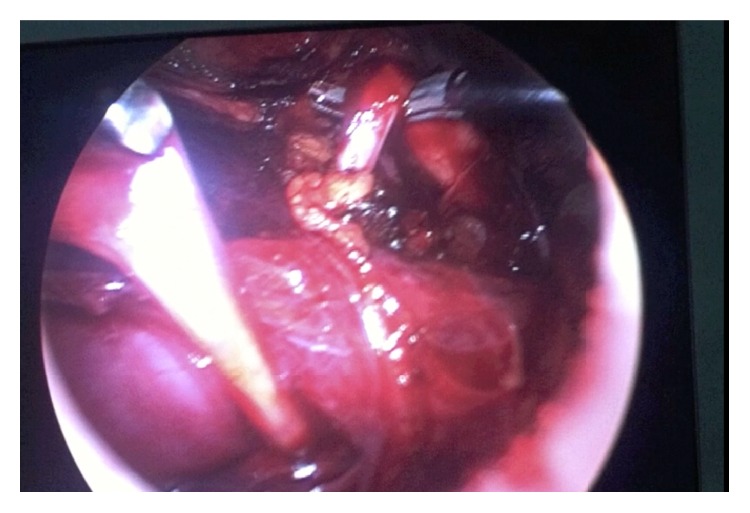
The ureter lifting up the vena cava like a surgical loop.

The colon was reflected medially exposing the retroperitoneum. The renal pelvis and proximal ureter were identified helped by the presence of the JJ catheter. The ureter was dissected and followed until the right side of the vena cava. In the interaortocaval area the ureter was identified and dissected caudally (Figures 2 and 3). Using sharp and blunt dissection the retrocaval segment was then entirely mobilized and separated from the inferior vena cava (IVC). The renal pelvis was sectioned. The stent was partially withdrawn and the ureter was drawn medially from behind the vena cava. After checking that the retrocaval portion was not atretic, the renal pelvis and the ureter were reanastomosed with running 4-0 polyglactin sutures in a normal anatomic position.

## 3. Results

All operations were achieved laparoscopically without conversion to open surgery. The mean operative time was 140 minutes (190, 110, and 120 minutes for patients 1, 2, 3, resp.).

No intraoperative vascular or digestive complication occurred. Blood loss was less than 50 mL in all patients. Intravenous paracetamol was administrated during the first postoperative day for pain control. No patient required opioid analgesia. The Foley catheter and the drain were removed 3-4 days postoperatively.

No postoperative complications occurred except one female patient who had in the early postoperative period irritative symptoms due to JJ stent and these were controlled with anticholinergic drugs. All patients were discharged 3 days after the operation and the mean hospital stay was 5 days (range, 4–6 days).

The JJ stent was removed 8–12 weeks postoperatively. After removing the stent, patients were followed clinically and by ultrasound every 3 months. All patients were symptom-free after surgery and had reduction of the hydronephrosis.

The control CT scan with urography performed 9 months after surgery in two patients demonstrated permeable upper urinary tract without stenosis.

## 4. Discussion

Retrocaval ureter is a rare congenital anomaly, with a reported incidence of 1/1000 live births [6, 7]. This incidence added to the vascular risk with the dissection of the vena cava and the technical difficulty to perform laparoscopic ureteral sutures explains the few reported cases of laparoscopic repair of this anomaly. When using MeSH terms, retrocaval ureter and laparoscopy, on PubMed, only 40 articles are found. To our knowledge, this is the unique African series.

Surgery is indicated when the disease is associated with symptoms or complication. This is usually the case in the third or fourth decade of life [8]. All our patients were symptomatic and the mean age was 36 years (range 18–48).

The first laparoscopic repair has been reported by Baba et al. who performed laparoscopic ureteroureterostomy in 9 h and 20 min with anastomosis time of 2.5 h using 5 laparoscopic ports [9]. Since that, with increasing experience and the improvement of instruments, some authors started to apply laparoscopy to the treatment of retrocaval ureter.

Both the transperitoneal and the retroperitoneal approach can be used [10].

Dogan et al. operated on 4 patients using the transperitoneal approach [11]. Mean operation time was 210 minutes. No intraoperative complications occurred. In one patient, antegrade double-J-stent placement failed, and the stent was therefore placed in the retrograde way.

Simforoosh et al. [8] reported a series of 6 cases of retrocaval ureter that were successfully treated with a transperitoneal laparoscopic approach. Mean operative duration was 180 minutes (range 150 to 210) and patients were discharged home at a mean of 4 days (range 3 to 5).

Ding et al. [12] reported the largest series of transperitoneal approach in 2012. Nine patients underwent pure laparoscopic pyelopyelostomy or ureteroureterostomy. The mean operative time was 135 minutes (range, 70–250 minutes). No intraoperative complications or significant bleeding occurred.

Other authors preferred retroperitoneal* * approach. Xu et al. used this way to treat retrocaval ureter [13]. Seven patients underwent retroperitoneal repair. The mean operating time was 128.6 minutes (range 97–189). The mean blood loss was 20 mL (range 15–50).

Li et al. [14] operated on a total of 10 patients. All operations were completed laparoscopically. The mean operative time was 82 minutes (range, 60–110 minutes) and the blood loss was minimal.

Chen et al. [15] reported the largest series of retroperitoneal laparoscopic ureteroureterostomy for retrocaval ureter with 12 patients operated on. No open conversion was needed. The mean operating time was 112 minutes (range 89–158), and the mean anastomosis time was 42 minutes.

None of the mentioned studies reported intraoperative complications or blood transfusion.

Our results are comparable to these series. All operations were achieved laparoscopically without conversion to open surgery. The mean operative time was 140 minutes (110–190). No intraoperative vascular or digestive complication occurred. Blood loss was less than 50 mL in all patients.

We believe that each technique (transperitoneal and retroperitoneal) has advantages and drawbacks. It seems that retroperitoneal access is less time-consuming probably because it provides direct access to the ureter and inferior vena cava. We opted for the transperitoneal route because it provides superior exposure and more working space and as a result of our limited experience with the retroperitoneal approach. Intracorporeal sutures may be easier by transperitoneal way than retroperitoneal one. In our opinion, working space is more limited by retroperitoneal access, and the dilation may cause haemorrhage that can affect visibility making the procedure more difficult.

Other arguments are reported by Ding et al. [12]:

- Urologists are more familiar with the method of operation and anatomical features.
- The closure of the peritoneum and Gerota's fascia after the anastomosis can reduce urine spillage into the peritoneal cavity.
- Unnecessary dissection can be avoided. After opening the peritoneum and Gerota's fascia, dissecting the anteromedial lower pole of the kidney is sufficient to expose the renal pelvis and upper ureter.
- Most of the kidney can keep its entirety and stay in the normal position without dropping. So we do not need the fourth port to assist the exposure.

Many authors consider laparoscopic suture as the most difficult and time-consuming step of the procedure. Ishitoya et al. [16] and Tobias-Machado et al. [17] proposed extracorporeal suture for ureteroureterostomy performed through minilaparotomy. With increasing experience and developing skills, and also with training programs available, this constraint will be surpassed. Another trick is to perform a pyelopyelostomy further than ureteroureteral anastomosis. Pyelopyelostomy is easier because it offers more space for grasping and passing the needle.

In our series, none of the patients had resection of the retrocaval ureter witch is compared to other series. This segment was not atretic and there is a risk of inducing high tension when performing anastomosis.

When comparing with other authors, we are the only authors to perform systematic retrograde JJ stent placement. This is a principle in our department when operating ureteropelvic junction obstruction and we applied it to retrocaval ureter. It permits realizing a RGP to avoid missing concurrent pathologies. Antegrade JJ stent placement can sometimes be challenging; it requires the use of C arm to confirm the presence of the lower curve of the JJ stent in bladder which is time-consuming. Some authors use methylene blue instilled in the bladder but this can spoil the surgical field and make the anastomosis more difficult. We believe, furthermore, that the placement of the catheter will facilitate the dissection and confirm that the retrocaval segment is not atretic.

To have a virtually scar-free result, Autorino et al. [10] described in 2010 the first successful case of laparoendoscopic single-site surgery (LESS) repair of retrocaval ureter. Operative time was 3 hours which is comparable to mean time with standard laparoscopy. From June 2010 to May 2011, Kang et al. [18] used the LESS procedure with retroperitoneal approach to operate on 4 patients with retrocaval ureter. The single-port device was made with a surgical glove and Foley catheter and allowed the introduction of three trocars. The mean operating time was 105 min (range, 90–135 min). None of the patients required blood transfusion. These results are encouraging but the real place of LEES remains debated. The authors have considered that LESS needs further refinement in instrumentation before the technique be considered as standardized as the conventional laparoscopy. Also, no benefit in terms of recovery has been demonstrated when comparing LESS nephrectomy and LESS pyeloplasty with the classic laparoscopy [19, 20].

More recently, some observations [21, 22] reported the application of robotic laparoscopy to the management of retrocaval ureter. Obviously, due to ergonomic benefit and 3-dimensional vision, this technology may improve surgeon dexterity and quality of dissection, but the problem of high procedure cost may be an obstacle especially in emerging countries.

A case of augmented reality techniques assisted laparoscopic ureteroureterostomy for retrocaval ureter has been reported by Wu et al. [23] in 2012. The author proposes this technique to overcome the problems due to the limitation of tactile feedback and loss of 3D visualization during laparoscopy and to minimize the risk of iatrogenic injury.

Though the number of cases in this study does not allow a definitive statement, it provides an idea about the place that could have laparoscopy in the management of retrocaval ureter. Larger series are needed to standardize the technique and compare the transperitoneal and retroperitoneal approaches.

## 5. Conclusion

Certainly our series is small, but knowing the advantages of laparoscopy as a minimally invasive technique with less bleeding, less drug requirement, and short hospital stay while performing various procedures for different diseases in urology, it should be proposed as a first-line treatment for retrocaval ureter. The choice of transperitoneal or retroperitoneal approach depends on the preferences of the surgeon.
